# A prospective investigation of injury incidence and risk factors among army recruits in combat engineer training

**DOI:** 10.1186/1745-6673-8-5

**Published:** 2013-03-05

**Authors:** Joseph J Knapik, Bria Graham, Jacketta Cobbs, Diane Thompson, Ryan Steelman, Bruce H Jones

**Affiliations:** 1 US Army Institute of Public Health, Epidemiology and Disease Surveillance Portfolio, ATTN: MCHB-IP-DI, 5158 Blackhawk Rd, Aberdeen Proving Ground, MD 21010, USA; 2 Hawaii Department of Health, Honolulu, HI, USA; 3 Department of Obstetrics and Gynecology, Medical University of South Carolina, Charleston, SC, USA; 4 AIDS Resource Center of Wisconsin, Milwaukee, WI, USA

**Keywords:** Age, Body mass index, Smoking, Exercise, Physical activity, Prior injury, Education

## Abstract

**Background:**

United States Army combat engineer (ENG) training is an intense 14-week course designed to introduce new recruits to basic soldiering activities, Army values and lifestyle, and engineering skills and knowledge. The present investigation examined injury rates and injury risk factors in ENG training.

**Methods:**

At the start of their training, 1,633 male ENG recruits were administered a questionnaire containing items on date of birth, height, weight, tobacco use, prior physical activity, and injury history. Injuries during training were obtained from electronic medical records and the training units provided data on student graduation and attrition. Risk factors were identified using Cox regression.

**Results:**

Ninety-two percent of the recruits successfully graduated from the course and 47% of the recruits experienced one or more injuries during training. Univariate Cox regression demonstrated that recruits were at higher injury risk if they reported that they were older, had a higher or lower body mass index, had smoked in the past, had performed less exercise (aerobic or muscle strength) or sports prior to ENG training, had experienced a previous time-loss lower limb injury (especially if they had not totally recovered from that injury), or had a lower educational level.

**Conclusions:**

The present investigation was the first to identify injury rates and identify specific factors increasing injury risk during ENG training. The identified risk factors provide a basis for recommending future prevention strategies.

## Background

The United States (US) Army Combat Engineer (ENG) course is a physically intense, 14-week course designed to develop basic soldiering skills, introduce Army values and lifestyle, and impart engineering knowledge and skills that are used in combat operations. The first 10 weeks of training involves basic soldiering and the recruit is very active with almost daily physical training (exercise) in addition to periodic road marches, obstacle course negotiation, marksmanship training, drill and ceremony, high tower operations, team and individual movement exercises, land navigation, and other physical activities. The latter weeks are devoted to training more specific to combat engineering. Physical training continues and other activities include operation of heavy equipment, construction of fighting positions, erection of obstacles and defensive positions, placement and detonation of explosives, route clearance of obstacles, use of fixed or floating bridges, preparation and installation of firing systems for demolition and explosives, and training in techniques to detect mines either visually or with mine detectors.

A number of previous studies have examined injury rates in Basic Combat Training (BCT) [1-5] and infantry one-station unit training (OSUT) [6] and we recently examined injury rates and injury risk factors in military police (MP) OSUT [7]. In this latter investigation we demonstrated that male recruits were at higher injury risk if they were older, had smoked in the past, had performed less frequent exercise or sports prior to MP training or had a prior injury [7]. Identifying injury risk factors in military training provides a basis for recommending future prevention strategies. The activities involved in the diverse types of military training may put personnel at different injury risks and thus it is important to investigate each type of training separately. The purpose of the present investigation was to examine injury rates and injury risk factors in ENG OSUT training.

## Methods

Participants were 1,633 men participating in ENG OSUT at Fort Leonard Wood, Missouri. These recruits were from 11 separate training companies in two battalions that began training between 9 August 2010 and 14 March 2011 and completed training between 19 November 2010 and 24 June 2011. None of the ENG OSUT companies involved in “Exodus” were included. Exodus was a two-week period over the late December and early January period when no training was conducted and the recruits were allowed to return to their homes. Ethical approval for the study was granted by Human Subjects Protection Office at the US Army Institute of Public Health [8] which follows the Helsinki Declaration on Ethical Principles for Medical Research Involving Human Subjects. An anonymised database was used for the analyses.

### Procedures

Recruits completed a lifestyle questionnaire within the first week of training. This questionnaire contained items on date of birth, height, weight, tobacco use, prior physical activity, and prior injury history. Recruit demographic data were obtained from the Defense Manpower Data Center (DMDC). The DMDC systematically collects and maintains an archive of military manpower, personnel, and training data to support military information management needs. Demographic information obtained from the DMDC for this investigation included component (active, reserve, National Guard), educational level, marital status, and race.

Recruit injuries were obtained from the Defense Medical Surveillance System (DMSS) of the Armed Forces Health Surveillance Center (AFHSC). The AFHSC regularly compiles data on ambulatory (outpatient) encounters occurring within military treatment facilities, as well as those occurring outside these facilities (civilian care) and paid for by the US Department of Defense. A list of recruits from the units being evaluated and the dates of their complete training cycles were provided to the AFHSC. The AFHSC returned visit dates and International Classification of Diseases, Revision 9, Clinical Modification (ICD-9) codes for all outpatient medical visits during the training cycle timeframe. Five injury indices were calculated from the data provided by the AFHSC. These indices were the Installation Injury Index (III), the Modified Installation Injury Index (MIII), the Training Related Injury Index (TRII), the Comprehensive Injury Index (CII), and the Overuse Injury Index (OII). These indices included specific ICD-9 codes, as described previously [9]. The III and TRII were developed by personnel at the AFHSC. The III has been used to compare overall injury rates (acute and overuse) among military posts and is reported on a monthly basis at the AFHSC website (http://afhsc.army.mil). The TRII is limited to lower extremity overuse injuries and has been used to compare injury rates among Army basic training locations. The MIII, CII, and OII were developed by personnel in the Injury Prevention Program at the AIPH. The MIII captures a greater number of injuries than the III, including more overuse-type injuries. The CII captures all ICD-9 codes related to injuries defined as physical damage to the body as a result of an energy exchange [10,11]. The OII captures the subset of musculoskeletal injuries presumably resulting from cumulative microtrauma (overuse injuries) such as stress fractures, stress reactions, tendonitis, bursitis, fasciitis, arthralgia, neuropathy, radiculopathy, shin splints, synovitis, sprains, strains, and musculoskeletal pain (not otherwise specified).

Recruits that attrited from training, as well as the date and reason, were provided by the training companies. These data were verified from information in the Directorate of Human Resources, Trainee Student Processing Branch at Fort Leonard Wood. Attrition could have been due to discharge from service or recycling. A discharge was a recruit who was not suitable for service in the Army and was formally released from their service commitment. A discharge could have been due to a medical condition that existed prior to service or developed during BCT, or for a non-medical reason. Non-medical discharges were generally due to the inability of the recruit to adapt to the military environment because of lack of ability (could not adequately perform critical military tasks) or for psychosocial reasons (lack of motivation, inability to follow orders, personality problems, commission of serious offenses). A recycle was a recruit who needed additional training to complete training requirements and was sent to another unit to complete this training. Recycles were not followed once they left their initial training unit.

### Data analysis

Age was calculated from the date of birth to the date of the start of training. Body mass index (BMI) was calculated as weight/height^2^ obtained from the questionnaire [12]. Cumulative injury incidence was calculated as the number of recruits with ≥1 injury/the total number of recruits × 100%. Injury incidence rate was calculated as the number of recruits with ≥1 injury/the total number of recruits × number of days in training (injuries/1,000 person-days).

Other analyses were performed using the Statistical Package for the Social Sciences (SPSS), Version 18.0. Cox regression (survival analysis) was used to examine the association between the time to the first CII injury and potential injury risk factors. Once a recruit had an injury, his contribution to time in training was terminated (censored). Those who attrited from training had their time censored at the day they left training, unless their time had already been censored as the result of an injury. All potential risk factors were entered into the regression models as categorical variables. Continuous variables were converted to categorical variables based on recommendations from the literature or findings from previous basic training investigations [5,13,14]. Age was separated into 4 groups (<20.0, 20.0-24.9, 25.0-29.9, and ≥30.0 years). BMI was separated into 4 groups (<18.5, 18.5-24.9, 25.0-29.9 and ≥30.0 kg/m^2^) as recommended by the National Institute of Health [15]. Physical activity questions were categorized based on American College of Sports Medicine recommended frequency of physical activity [16]. For all Cox regressions, simple contrasts were used, comparing the injury hazard at a baseline stratum of a variable (defined with a hazard ratio (HR) of 1.00) with other strata of the same variable. Variables were included in a multivariate backward stepping Cox regression if they achieved p < 0.10 in the univariate analyses [17]. Multivariate Cox regressions established the association between a variable and injury risk while controlling for other significant injury risk factors.

## Results

The mean ± standard deviation age, height, weight, and BMI of the recruits was 21.6 ± 3.9 years, 177 ± 7 cm, 78 ± 12 kg, and 24.9 ± 3.4 kg/m^2^, respectively. Most recruits graduated (91.5%, n = 1,494) but 3.0% (n = 49) received medical discharges, 3.6% (n = 58) received other than medical discharges, 1.9% (n = 31) were recycled, and 0.1% (n = 1) went absent without leave.

Table 1 shows the injury incidence and injury incidence rates for each of the injury indices. The entire cohort contributed a total of 153,250 person-days of risk.

**Table 1:** Injury incidences and injury incidence rates in combat engineer training

**Injury index**	**Injury incidence (%)**	**Injury incidence rate (injuries/1,000 person-days)**
Installation injury index	44.2	4.64
Modified installation injury index	46.5	4.88
Overuse injury index	38.1	4.00
Training-related injury index	31.1	3.27
Comprehensive injury index	46.7	4.91

Table 2 displays the univariate associations between injury risk and the variables under investigation. Not all recruits answered all questions so the sample sizes are shown. Higher injury risk was associated with older age, higher and lower BMI, having smoked ≥100 cigarettes in the past, smoking ≥20 cigarettes in the 30 days before ENG training, a lower level of physical activity compared to peers, less frequent exercise/sports, less frequent running/jogging, less frequent weight training, a shorter history of running/jogging or weight training, a prior lower limb injury (especially if that injury prevented activity for ≥1 week or the recruit had not totally recovered from the injury) and a lower level of educational attainment.

**Table 2:** Univariate associations between variables and injury risk among combat engineer recruits

**Variable group**	**Variable**	**Strata**	**N**	**Injury incidence (%)**	**Hazard ratio (95% CI)**	**p-value (Wald statistic)**
Age	Age	<20.0 years	722	44.3	1.00	Referent
		20.0-24.9 years	690	46.7	1.12 (0.96-1.31)	0.14
		25.0-29.9 years	144	50.7	1.30 (1.01-1.68)	0.04
		≥30.0 years	77	62.3	1.81 (1.34-2.46)	<0.01
BMI	BMI	<18.5 kg/m^2^	27	74.1	2.32 (1.48-3.64)	<0.01
		18.5-24.9 kg/m^2^	845	46.9	1.00	Referent
		25.0-29.9 kg/m^2^	628	43.5	0.93 (0.80-1.09)	0.37
		≥30 kg/m^2^	127	54.3	1.38 (1.07-1.78)	0.02
Prior tobacco use	Smoked ≥100 cigarettes in life	No	1000	44.8	1.00	Referent
		Yes	632	49.8	1.17 (1.01-1.35)	0.04
	Age started smoking	Never smoked	745	46.7	1.00	Referent
		<13 years	84	50.0	1.08 (0.79-1.49)	0.63
		13-16 years	550	47.5	1.02 (0.87-1.20)	0.81
		≥17 years	254	44.2	0.92 (0.75-1.14)	0.47
	Days smoked in 30 days before ENG training	None	1098	46.0	1.00	Referent
		1-9 days	146	45.9	1.00 (0.77-1.29)	0.99
		10-19 days	92	46.7	1.00 (0.74-1.37)	0.98
		≥20 days	297	49.8	1.11 (0.92-1.33)	0.27
	Cigarettes smoked in 30 days before ENG training	None	1112	46.2	1.00	referent
		1-9 cigarettes/day	303	47.2	1.00 (0.83-1.21)	0.98
		10-19 cigarettes/day	146	45.2	0.98 (0.76-1.26)	0.86
		≥20 cigarettes/day	71	54.9	1.36 (0.98-1.88)	0.07
	Days of smokeless tobacco use in 30 days before ENG training	None	1331	48.5	1.00	Referent
		1-9 days	72	37.5	0.73 (0.50-1.07)	0.11
		10-19 days	61	38.4	0.74 (0.41-1.05)	0.14
		≥20 days	168	48.7	1.01 (0.61-1.32)	0.12
	Amount of smokeless tobacco use in 30 days before ENG training	None	1341	48.2	1.00	Referent
		≤3/4 cans, plugs	125	41.2	0.84 (0.67-1.05)	0.14
		1 to 1-3/4 cans, plugs	121	41.7	0.84 (0.69-1.09)	0.11
		≥2 cans, plugs	43	51.2	1.16 (0.76-1.77)	0.50
Prior physical activity	Physical activity before ENG training, compared to other of same age and sex	Much less active	70	67.1	2.46 (1.75-3.47)	<0.01
		Less active	293	59.7	1.83 (1.44-2.33)	<0.01
		Average	421	42.8	1.12 (0.88-1.42)	0.38
		More active	583	43.7	1.13 (0.90-1.42)	0.29
		Much more active	x265	40.0	1.00	Referent
	Exercise or sports frequency 2 months before ENG training	≤1 time/week	187	58.3	1.76 (1.40-2.22)	<0.01
		2-4 time/week	907	48.2	1.26 (1.07-1.49)	<0.01
		≥5 time/week	538	40.3	1.00	Referent
	Running/jogging Frequency before ENG training	≤1 time/week	478	52.3	1.46 (1.15-1.86)	<0.01
		2-4 times/week	926	45.6	1.17 (0.93-1.47)	0.17
		5-7 times/week	227	39.6	1.00	Referent
	Time running/jogging before ENG training	≤1 month	484	50.4	1.38 (1.10-1.72)	<0.01
		2-6 months	876	46.6	1.19 (0.97-1.47)	0.11
		≥7 months	273	40.7	1.00	Referent
	Weight training frequency before ENG training	≤1 time/week	646	51.5	1.28 (1.04-1.57)	0.02
		2-4 times/week	699	43.8	0.99 (0.81-1.22)	0.95
		5-7 times/week	286	43.4	1.00	Referent
	Time weight training before ENG training	≤1 month	671	50.7	1.30 (1.08-1.56)	<0.01
		2-6 months	544	45.2	1.12 (0.92-1.36)	0.26
		≥7 months	417	42.2	1.00	Referent
Prior injury	Prior lower limb injury	No	1234	45.3	1.00	Referent
		Yes	399	51.1	1.21 (1.03-1.43)	0.02
	Prior injury prevent activities ≥1 week	No prior injury	1234	45.3	1.00	Referent
		No	131	47.3	1.04 (0.80-1.36)	0.76
		Yes	267	52.8	1.30 (1.08-1.57)	<0.01
	Totally recovered from prior injury	No prior injury	1229	45.2	1.00	Referent
		No	18	61.1	2.17 (1.19-3.93)	0.01
		Yes	380	50.5	1.18 (1.00-1.39)	0.05
Demo-graphics	Component	Active Army	972	47.8	1.00	Referent
		National Guard	463	45.1	0.89 (0.76-1.05)	0.17
		Army Reserve	198	44.9	0.96 (0.76-1.20)	0.69
	Educational level	<High school graduate	99	57.6	1.39 (1.05-1.82)	0.02
		High school graduate	1315	46.3	1.00	Referent
		Some college	150	46.0	1.01 (0.79-1.30)	0.93
		≥College graduate	52	40.4	0.91 (0.59-1.40)	0.66
		Unknown	17	41.2	0.83 (0.40-1.75)	0.63
	Race/ethnicity	White	1122	45.5	1.00	Referent
		Black	239	49.0	1.09 (0.89-1.34)	0.39
		Hispanic	191	49.2	1.08 (0.87-1.34)	0.50
		Other	78	53.8	1.32 (0.96-1.81)	0.09
		Missing	3	0.0	^a^	^a^
	Marital status	Single, never married	1380	50.0	1.00	Referent
		Married	238	46.1	1.16 (0.96-1.42)	0.13
		Other	15	53.3	1.16 (0.58-2.33)	0.68

^a^Not included in the analysis.

Table 3 shows the results of the multivariate Cox regression. Multivariate Cox regression requires complete data on all variables and this was available on 1,620 recruits (99%). Independent injury risk factors included older age, lower BMI, a lower physical activity self-rating, reporting not having recovered from a previous injury and a lower level of educational attainment.

**Table 3:** Multivariate association between variables and injury risk among combat engineer recruits

**Variable**	**Strata**	**N**	**Hazard ratio (95% CI)**	**p-value**
Age	<20.0 years	716	1.00	Referent
	20.0-24.9 years	684	1.16 (0.98-1.36)	0.08
	25.0-29.9 years	143	1.48 (1.13-1.94)	<0.01
	≥30.0 years	77	2.36 (1.69-3.28)	<0.01
BMI	<18.5 kg/m2	27	2.39 (1.52-3.75)	<0.01
	18.5-24.9 kg/m^2^	844	1.00	Referent
	25.0-29.9 kg/m^2^	623	0.86 (0.73-1.00)	0.06
	≥30 kg/m^2^	126	1.16 (0.90-1.51)	0.26
Physical activity before basic training compared to peers	Much less active	70	2.46 (1.73-3.49)	<0.01
	Less active	289	1.85 (1.45-2.37)	<0.01
	Average	418	1.15 (0.90-1.47)	0.28
	More active	579	1.13 (0.90-1.42)	0.30
	Much more active	264	1.00	Referent
Totally recovered from prior injury	No prior injury	1226	1.00	Referent
	No	18	2.21 (1.20-4.08)	0.01
	Yes	376	1.15 (0.98-1.36)	0.09
Educational level	<High school graduate	98	1.43 (1.09-1.89)	0.01
	High school graduate	1305	1.00	Referent
	Some college	149	0.94 (0.73-1.22)	0.66
	≥College graduate	51	0.62 (0.39-1.00)	0.05
	Unknown	17	0.86 (0.41-1.82)	0.70

## Discussion

The present investigation was the first to quantify the injury risk and identify injury risk factors in ENG training, and one of the few [6,7] to explore these issues in any type of OSUT training. Past investigations of infantry [6] and MP OSUT [7] that used a definition of injury similar or identical to that of the present investigation [7] found injury incidences of 46% and 34%, respectively. This compares with the incidence of 47% in the present study. The high injury incidence in combat engineer and infantry training likely reflect the intensity and variety of physical activities in these occupational specialties that put the recruits at risk of injury. Previous studies in basic training [18,19] and studies in the civilian sector [20,21] have shown that as physical activity increases so does the incidence of injuries.

In addition to documenting injury incidence, the present investigation identified a number of factors that put recruits at higher injury risk. Older age was an independent injury risk factor among both men and women and this is in consonance with other studies in BCT [2,5,6,22,23] and MP OSUT [7], as well as other military and civilian investigations where participants performed similar levels of physical activity [24-26]. Of interest was the fact that during the time of this study the age requirement for entry into the Army had been liberalized to allow into service individuals 17 to 45 years of age, whereas previously it had been 17 to 35 years. The present investigation had 31 individuals over the age of 35 and this made up only 1.9% of the entire cohort, but 40% of those over the age of 30 years.

The reason for the higher susceptibility to injury in older recruits may have to do with age-related changes in stem cells that slow tissue healing [27-29], age-related declines in fitness [30,31] since lower fitness has been shown to be associated with injuries [1,2,22,32-35] and/or prior injury history since older recruits may be more likely to have experienced lower limb injuries in the past that make them more susceptible to injuries during ENG training [33,36-39]. To examine if older recruits were more likely to report a prior injury in the present investigation, self-reported prior lower limb injuries were stratified by age. Table 4 shows that younger and older age groups generally had similar differences in injury incidence so the hypothesis was not supported here. This is similar to findings in other investigations [7,26].

**Table 4:** Injuries in combat engineer training stratified by prior lower limb injury and age

	**Response category**	**N**	**Injured in ENG training (%)**	**Risk ratio –prior/no prior injury (95% CI)**	**p-value**^**a**^
<20.0 year olds	No prior injury	552	42.9	1.14 (0.95-1.36)	0.18
	Prior injury	170	48.8		
20.0-24.9 year olds	No prior injury	522	45.0	1.15 (0.97-1.37)	0.13
	Prior injury	168	51.8		
25.0-29.9 year olds	No prior injury	107	51.4	0.95 (0.65-1.38)	0.77
	Prior injury	37	48.6		
≥30 year olds	No prior injury	53	60.4	1.10 (0.77-1.58)	0.60
	Prior injury	24	66.7		

^a^Chi-square statistic.

Beside older age, the present study found that recruits with either high or low levels of BMI had higher injury risk compared to those of “normal” BMI, although in the multivariate analysis the risk at the highest BMI level (“obese”) was reduced compared to the univariate result. Most basic training studies have reported bimodal relationships [1,22,35,40], although a few [2,41] have not, or have reported increased risk with only high [42] or low [43] BMI. The latter two studies [42,43] had a very narrow range of BMIs perhaps making it difficult to demonstrate a bimodal relationship. High BMI generally indicates a larger percentage of body fat [12,44], although it is also possible to have a high BMI as a result of a higher amount of fat-free mass [45]. Low BMI may reflect a paucity of either fat, fat-free mass, or both. Low BMI may make recruits more susceptible to injury if they lack the muscle mass or strength in the supportive structures (ligaments, bones) required to perform certain physical tasks and/or if they overexert or overuse the available muscle mass or supportive structures leading to injuries. On the other hand, injury risk might be increased among those with high BMI because the additional mass would increase the intensity of physical activity [46] leading to more rapid fatigue and impose a higher volume of repetitive stress on the musculoskeletal system because of the greater weight relative to height. Compared to BCT or MP training [1,7,40], these factors may especially exacerbate injuries in ENG training because of the types of physical activities that ENGs perform, especially in activities involving construction of fighting positions and erection of obstacles.

In the present investigation, recruits who reported smoking ≥100 cigarettes in the past were at higher injury risk and there was also a trend such that those who had smoked ≥20 cigarettes per day were at increased injury risk. Cigarette smoking was not an independent injury risk factor in the multivariate analysis. Nonetheless, cigarette smoking prior to basic training has consistently been associated with increased injury risk in US Army and Air Force basic training [2,6,34,40,47], MP OSUT training [7], and in the basic training of other countries [22,48]. Further, smoking was associated with injury in infantry soldiers [49] and in other occupational groups [50-52]. The association between smoking and injuries has biological plausibility, both from a physiological and psychosocial perspective. There is considerable literature showing that cigarette smoking impairs wound [53,54] and bone [55-57] healing, reduces tissue strength [58,59], and affects immune function. The immune system is important for tissue healing, since macrophages, leukocytes, and lymphocytes regulate various steps in the process and remove or assist in removal of damaged tissue [60-63], such as might be produced by repetitive microtrauma. Besides physiological mechanisms, psychosocial factors may also be a consideration. Prior studies show that Air Force recruits [64] and civilians [65-67] who were cigarette smokers had higher scores than nonsmokers on various measures of risk taking. It is possible that the higher risk-taking behavior of smokers manifests itself in the activities of basic training and results in a higher injury rate among smokers.

In basic training, all recruits are required to cease smoking at the beginning of training and any mechanism proposing to account for the association between smoking and injuries must take this into account. Evidence for the longer-term effects of smoking comes from studies on collagen metabolism and other studies on immune function. One study [68] found derangements in collagen metabolism among smokers. Investigators followed weekly urinary hydoxyproline/creatine levels (a marker of collagen metabolism) from individuals 14 weeks after they had ceased smoking. It was estimated (by mathematical modeling) that hydoxyproline/creatine levels would return to the level of nonsmokers in about 71 weeks, among those who had previously smoked ≤ 40 cigarettes/day, while it would take 120 weeks to reach the same level in those who had been smoking > 40 cigarettes/day. Immune studies suggest that smoking-induced leukocytosis slowly decreases over time once smoking ceases [69-75]. One day to 6 weeks after smoking cessation, the leukocyte count was still elevated [71,75]. Three months after smoking cessation, the neutrophil concentration tended to decrease [70]. Leukocyte counts approached the level of nonsmokers the longer it had been since the individual stopped smoking, but men who had quit smoking for 10 years or more still had higher leukocyte counts than nonsmokers in one study [72]. Another investigation showed that men and women who had quit smoking for an average of 11 years had counts similar to those who had never smoked [69].

There were 6 physical activity items on the questionnaire. These were designed to provide 1) a single global assessment of physical activity (self-rating compared to peers) 2) three questions on the frequency of recent (last 2 months before OSUT) physical activity (one general question, one specific to aerobic training and one specific to weight training), and 3) two questions on the history of aerobic or strength training. Results from all six questions generally indicated that a lower frequency or shorter history of physical activity was associated with higher injury risk in a generally dose-dependent manner. These data are in consonance with previous studies of military basic training that found increased risk of injury among those who self-reported less physical activity [1,2,6,14,76,77]. In ENG training, recruits perform weight-bearing physical activity primarily in the form of standing in formation, walking, and running. It seems reasonable that a higher frequency of weight-bearing physical training prior to training would result in less susceptibility to injury because of the favorable influences of physical activity on the body. Physical activity of the proper intensity, frequency, and duration can increase aerobic fitness, muscle strength, connective tissue, bone strength, general health, and can reduce body fat [16,78-84]. These and other factors may explain the reduced susceptibility to injury among recruits who were physically active prior to ENG training [85].

Recruits who reported a prior lower limb injury were at higher injury risk. This relationship appeared to be graded, depending on the reported severity of the previous injury. That is, recruits reporting at least a week-long limitation of the previous injury were at higher risk in training than those who had previous injury but did not report a week-long limitation; those who reported that they had not totally recovered from the previous injury where at much higher risk than those with a prior injury who had recovered. Other studies of basic training have not demonstrated a consistent relationship between prior injuries and injuries in training [5-7,14,86], although this relationship has often been demonstrated in athletes [39,41,87-92]. Some authors have speculated that contractile or connective scar tissues may alter movement mechanics, or that muscle tissue atrophy induced by some injuries might reduce strength or result in muscle imbalances that could affect injuries [93,94]. Many injuries may be chronic or recurrent, accounting for at least a part of this relationship.

A lower level of education was an injury risk factor in both the univariate and multivariate analyses and a dose–response relationship was evident such that risk increased from high to low education. In consonance with the finding here, other investigations have found a graded relationship between injury-related morbibidity/mortality and educational attainment and/or various measures of intelligence in both military [95,96] and civilian [97,98] studies. Greater educational attainment may be associated with behaviors conducive to injury prevention [99] and/or the ability to more rapidly and effectively process information relating to risk reduction.

One of the limitations to the present study was the lack of prospective measures of physical fitness. Physical fitness has consistently been shown to be an independent risk factor for injuries in past investigations [1,2,5,13]. The Army Physical Fitness Test (APFT) was administered to recruits but it was given two to three weeks after the start of training. Since several physical training sessions were likely to have occurred since the start of training, and since recruits were likely to have considerably increased their physical fitness in this time, the first APFT could not serve as a baseline level of fitness.

## Conclusions

This paper has identified a number of key risk factors that can be used to target and suggest future prevention strategies for this and similar young, healthy populations. The multivariate model identified several important risk factors for injuries in this population including older age, lower body mass index, low prior physical activity levels, inadequate recovery from past injuries, and lower educational level. While older age per se as a risk factor cannot be modified, the degree of risk may be modifiable by starting training at lower levels and increasing training it more slowly as recommended for other at risk populations [100,101]. In regard to BMI, the Army has traditionally focused on screening for underweight soldiers and the focus on overweight has only been in place since 1960 [102]. The data presented in this paper suggest that consideration be given to underweight recruits, perhaps in terms of screening and/or increasing muscle mass and fitness levels. The excess risk of injury for recruits who reported low levels of physical activity prior to entering the Army is consistent with past studies and might be mitigated by initiating training at lower intensities and amounts followed by a more gradual progression of training as previously suggested [85,101]. Regarding injuries, it is not surprising that inadequate recovery from past injuries is a risk factor for future injuries. In the absence of stronger evidence, common sense dictates that soldiers who have sustained injuries should be given adequate time to recover and training should be structured to reduce pain and physiologic strain on the injured part [101]. The Army should continue its focus on recruiting those with higher educational level. In summary, findings from this study clearly show that modifiable risk factors can be identified by systematic research and used to recommend prevention strategies for injuries resulting from vigorous physical training activities, such as Army ENG training.

## Abbreviations

US: United States;ENG: Engineer;MP: Military police;BCT: Basic combat training;OSUT: One station unit training;DMDC: Defense manpower data center;DMSS: Defense medical surveillance system;AFHSC: Armed forces health surveillance center;ICD-9: International classification of diseases, revision 9, clinical modification;III: Installation injury index;MIII: Modified installation injury index;TRII: Training related injury index;CII: Comprehensive injury index;OII: Overuse injury index;BMI: Body mass index;HR: Hazard ratio;95% CI: 95% confidence interval;APFT: Army physical fitness test

## Competing interests

The authors have no competing interests.

## Authors’ contributions

JJK participated in the design of the study, assisted with data collection, assisted with compiling the data, performed the statistical analysis, interpreted the data, and drafted the manuscript. BG, JC, DT, RS collected and complied data, assisted with the interpretation of the data, and helped draft the manuscript. BHJ participated in the design of the study, assisted in the interpretation of the data, and helped draft the manuscript. All authors read and approved the final manuscript.

## Authors’ information

The views, opinions, and/or findings contained in this report are those of the authors and should not be construed as official Department of the Army position, policy, or decision, unless so designated by other official documentation. Approved for public release; distribution is unlimited.
